# Associations Between Alcohol Consumption and Cigarette Smoking and Insulin Secretion and Resistance in Vietnamese Men Without a History of Diabetes: A Cross-Sectional Study

**DOI:** 10.1155/jdr/1068375

**Published:** 2025-09-24

**Authors:** Sumiyo Okawa, Hiroyasu Iso, Yuta Yokobori, Masahiko Hachiya, Chau Que Nguyen, Thuy Thi Phuong Pham, Danh Cong Phan, Hung Thai Do, Tetsuya Mizoue, Yosuke Inoue

**Affiliations:** ^1^Bureau of Global Health Cooperation, Japan Institute for Health Security, Shinjuku, Tokyo, Japan; ^2^Institute for Global Health Policy Research, Bureau of Global Health Cooperation, Japan Institute for Health Security, Shinjuku, Tokyo, Japan; ^3^Department of Non-Communicable Disease Control and Nutrition, Pasteur Institute in Nha Trang, Nha Trang, Khánh Hòa, Vietnam; ^4^Pasteur Institute in Nha Trang, Nha Trang, Khánh Hòa, Vietnam; ^5^Department of Epidemiology and Prevention, Japan Institute for Health Security, Shinjuku, Tokyo, Japan

**Keywords:** alcohol drinking, insulin, tobacco smoking

## Abstract

**Introduction:** This study is aimed at examining the associations of alcohol consumption and cigarette smoking with insulin secretion and resistance and the effect modification by body mass index (BMI) in middle-aged men without a history of diabetes in rural Vietnam.

**Materials and Methods:** The study included data from 1102 men without diabetes, aged between 40 and 60 years, who participated in the Khánh Hòa Cardiovascular Study conducted between June 2019 and 2020. Fasting blood samples were collected to measure insulin and plasma glucose. Alcohol consumption was categorized as no consumers or consumers (< 1, 1–1.9, and ≥ 2 standard drinks per day). Smoking was categorized as never, former, and current smokers (≤ 10 or ≥ 11 cigarettes per day). The homeostatic model assessment (HOMA) was used to assess insulin secretion (HOMA-beta) and insulin resistance (HOMA-IR). A multilevel linear regression model was used to examine the associations of alcohol consumption and smoking with HOMA-beta and HOMA-IR. Effect modification by BMI was tested using the likelihood ratio test, and a set of stratified analyses according to BMI (23 kg/m^2^) was performed.

**Results:** Alcohol consumption showed a dose–response association with low HOMA-beta in both the unadjusted and BMI-adjusted models. Cigarette smoking was not associated with HOMA-beta in either the unadjusted or BMI-adjusted models. Alcohol consumption was not associated with HOMA-IR when BMI was unadjusted, but the highest alcohol consumer group showed a significantly lower HOMA-IR after adjustment for BMI. Current smoking was significantly associated with lower HOMA-IR when BMI was unadjusted, which association became marginally significant after adjusting for BMI.

**Conclusions:** Alcohol consumption was associated with reduced insulin secretion. Smoking was marginally associated with lower insulin resistance, potentially mediated by low BMI. Longitudinal studies are required to explore the mechanisms underlying the association between alcohol consumption, smoking, and glucose metabolism.

## 1. Introduction

Diabetes mellitus (DM) is a major chronic disease with an estimated global age-standardized prevalence of 6.1% in 2021 [1]. Various studies have examined the associations between lifestyle-related factors and DM to reduce the disease burden including factors such as alcohol consumption and cigarette smoking [2–6]. A meta-analysis of 38 studies reported a lower risk of DM in moderate alcohol drinkers (< 63 g per day) than in abstainers and an increased risk in heavy alcohol drinkers (above the threshold of 63 g per day) [4]. Another meta-analysis of 25 prospective cohort studies reported a 1.44 times higher risk of DM in current smokers than in nonsmokers [6].

To better understand the mechanisms underlying these associations, epidemiological studies have examined the association between lifestyle parameters and the two features that affect the development of DM: insulin secretion and resistance. For example, lower insulin secretion [7–12] and lower insulin resistance [13–15] have been reported in alcohol drinkers than in nondrinkers. The association between cigarette smoking and insulin secretion is inconsistent. Some studies have reported decreased insulin secretion [8, 16–19], some have reported increased insulin secretion in smokers [20], and other studies have reported no association [21, 22]. Furthermore, the association between cigarette smoking and insulin resistance remains controversial, with reports of increased insulin resistance [20, 23], decreased insulin resistance [16, 21], or no association [17, 19, 22, 24, 25].

From our literature review, we identified two research gaps. First, although body mass index (BMI) may act as both a mediator and an effect modifier for the association of alcohol consumption and cigarette smoking with insulin secretion and resistance, yet previous studies have not extensively explored this aspect. For example, although low-to-moderate alcohol consumption is associated with improved insulin sensitivity [13–15], it has been suggested to negatively affect individuals with low BMI who inherently have low insulin secretion capacity [12], even at modest levels (i.e., effect modification). Additionally, higher alcohol consumption can lead to weight gain, which may increase the risk of diabetes (i.e., mediation). Furthermore, smoking is known to contribute to weight loss [26–28] by increasing energy expenditure [29, 30], which may be related to that individuals with a low BMI have lower insulin resistance and secretion [9, 12, 13, 31] (i.e., mediation).

Second, evidence on these associations is limited to high-income countries. The pathogenesis of insulin impairment varies among racial and ethnic groups. Specifically, the Asian population has reduced insulin secretion and a lower BMI than the Caucasian population [32, 33]. In addition, the relationship between alcohol consumption, smoking, and glucose metabolism has been inadequately evaluated in public health and clinical settings in Vietnam, despite the high prevalence of alcohol abuse (15%), cigarette use (47%), and DM (5%) in adult men [34]. Therefore, there is an urgent need to develop evidence-based policies regarding alcohol consumption, cigarette smoking, and glucose metabolism.

This study is aimed at examining the associations of alcohol consumption and cigarette smoking with two measures of glucose metabolism (i.e., insulin secretion and resistance) in men without a history of DM in Vietnam, accounting for the effect modification by BMI on the associations. We also aimed to examine the associations between the two risk factors and fasting glucose as a sensitivity analysis to assess whether these associations corresponded to the results of the associations between alcohol consumption, cigarette smoking, insulin secretion, and resistance.

## 2. Materials and Methods

### 2.1. Study Design and Setting

Data for this study were obtained from the baseline survey of the Khánh Hòa Cardiovascular Study (KHCS) conducted in middle-aged residents living in Khánh Hòa Province, Vietnam. This province is located in the south-central coastal region of the country. Eight communes within a predominantly rural district were selected as study sites. A baseline survey was conducted between June 2019 and June 2020 [35].

### 2.2. Study Participants

Study participants of the KHCS were aged between 40 and 60 years at the time of recruitment and resided at the study sites for at least 6 months. Eligible participants were identified based on the resident list developed by the community health center staff, and recruitment continued until completing a sample size of 3000 individuals (men 1160 and women 1840) by convenience sampling. In the present study, we included men who had never been diagnosed with DM and excluded those who had a history of DM to avoid reverse causation, such as the effect of blood glucose control on lifestyle modifications and improved study outcomes following DM diagnosis. Six men with missing household income data were excluded. Women were excluded because they had a low prevalence of alcohol consumption (< 3%) and smoking (< 1%).

### 2.3. Data Collection

The survey consisted of face-to-face interviews using a structured questionnaire and anthropometric and biochemical measurements of blood. The survey collected information on socioeconomic characteristics (e.g., age, educational attainment, marital status, occupation, monthly household income, and parents' history of diabetes), lifestyle characteristics (alcohol consumption, cigarette smoking, fruit and vegetable consumption, and physical activity), and history of DM diagnosis and treatment.

The anthropometric measurements included height (centimeter) and weight (kilogram). The height and weight of the participants wearing light clothes were measured by trained workers using a portable stadiometer (Charder, HM200P, Tokyo, Japan) and a digital weight scale (Tania, HD-661, Tokyo, Japan), respectively, and anthropometric values were recorded to the first decimal place. Biochemical measurements included fasting plasma glucose and insulin, for which participants were requested to fast for 8 h before blood collection. Blood samples were collected via venipuncture and centrifuged onsite. The samples were stored at a temperature below 4°C and transported to the Pasteur Institute in the Nha Trang laboratory. Fasting plasma glucose and insulin levels were measured using a Cobas 8000 analyzer (Roche, Switzerland).

### 2.4. Exposure Variable: Alcohol Consumption and Cigarette Smoking

Based on the self-reported information, alcohol consumption was classified into four categories as nonconsumers, consumers < 1, 1–1.9, and ≥ 2 standard drinks per day (i.e., a standard drink contains approximately 10 g of ethanol according to the World Health Organization's [WHO] STEPS surveillance) [36]. Cigarette smoking was divided into four categories as never smokers, former smokers, and current smokers who smoked ≤ 10 or ≥ 11 cigarettes per day.

### 2.5. BMI

BMI was calculated by dividing the weight (kilogram) by height squared (meter squared). Based on WHO classification of BMI for Asian adults, we categorized the participants into four groups (< 18.5 kg/m^2^ [underweight], 18.5–22.9 kg/m^2^ [normal range], 23.0–24.9 kg/m^2^ [overweight], 25.0–29.9 kg/m^2^ [Obese 1], and ≥ 30 kg/m^2^ [Obese 2]) [37]. For the stratified analyses, we divided the study participants at a BMI cutoff range of 23 kg/m^2^ (BMI < 23; ≥ 23 kg/m^2^), which was the closest cutoff to the median BMI for our study participants (i.e., 22.5 kg/m^2^).

### 2.6. Outcome Variables: Insulin Secretion and Resistance

The homeostasis model assessment indices for insulin secretion (HOMA-beta) and insulin resistance (HOMA-IR) were calculated using fasting glucose and insulin levels. HOMA-beta was calculated as (20 × fasting insulin [microunits per liter])/(fasting glucose [millimoles per liter] − 3.5), and HOMA-IR was calculated as (fasting insulin [microunits per liter] × fasting glucose [millimoles per liter])/22.5 [38]. Two participants with HOMA-beta values lower than zero were replaced with zero. Subsequently, 0.01 was added to the values for all participants. HOMA-beta and HOMA-IR values were logarithmically transformed to achieve a normal distribution of the values.

### 2.7. Covariates

Sociodemographic and economic factors that were used as variables in the adjusted models included age (40–44 years, 45–49 years, 50–54 years, and 55–60 years), education (primary school or lower, secondary school, and senior high school or higher), occupation (government employee, nongovernment employee, self-employed, farmer or fisherman, unemployed or housework, and other), diabetic history of parents (yes, no or unknown), and tertiles of the equivalent household income that is monthly household income divided by the square root of the number of household members (low [median 2,474,874 VND], middle [median 4,472,136 VND], and high [median 7,071,068 VND]) ($1= 23,475 VND as of June 1, 2019). For participants who were not the head of the household, income information provided by the head of their household was used. Lifestyle variables used in adjusted models included fruit and vegetable consumption (< 1, 1–1.9, 2–2.9, and ≥ 3 servings per day) and physical activity estimated by the metabolic equivalent of task (MET) based on the WHO's Global Physical Activity Questionnaire (< 600 and ≥ 600 METs minutes per week) [39].

### 2.8. Statistical Analysis

Descriptive analysis was performed to examine the distribution of sociodemographic, economic, lifestyle, and anthropometric characteristics according to the alcohol consumption and cigarette smoking categories.

Multilevel linear regression was performed to examine the association of alcohol consumption and cigarette smoking categories with log-transformed HOMA-beta and log-transformed HOMA-IR and estimated coefficient (Coeff.) with 95% confidence intervals (95% CIs). The geometric means (GMs) of HOMA-beta and HOMA-IR per category of the two exposure variables were estimated based on the postestimation of multilevel linear regression models. We employed four models, with never smokers and nonalcohol consumers serving as reference groups. Models 1a and 1b included alcohol consumption and cigarette smoking, respectively. Model 2 included the two exposure variables and age. Model 3 included education, occupation, household income, parental history of diabetes, fruit and vegetable consumption, and physical activity in addition to the variables included in Model 2. We considered Model 3 as the main model which comprised the effect of BMI as a mediator. Model 4 included BMI in addition to the variables included in Model 3, which controlled for the effect of BMI. *p* values < 0.05 were considered statistically significant, and those < 0.10 were considered marginally significant.

Furthermore, the effect modification by BMI was tested using the likelihood ratio test based on the analyses of Model 4. *p* values for the likelihood ratio test < 0.20 were considered suggestive of effect modification, leading to subsequent stratified analyses. Then, we performed a set of stratified analysis that divided the participants based on a BMI of 23 kg/m^2^ (BMI < 23 and ≥ 23 kg/m^2^) to control for BMI as an effect modifier.

Additionally, we examined the association between two exposures (i.e., alcohol consumption and smoking status) and fasting glucose levels to determine how these exposures were associated with fasting glucose levels as a result of their associations with insulin resistance and secretion. Fasting glucose levels were converted from millimoles per liter to milligrams per deciliter by multiplying the original values by 18.018, followed by logarithmic transformation to achieve a normal distribution. All statistical analyses were performed using Stata 17.0 (StataCorp, College Station, Texas, United States).

### 2.9. Ethical Considerations

We obtained approval from the ethics committees of Pasteur Institute in Nha Trang (Approval Number 02/2019/HĐĐĐ-IPN) and the National Center for Global Health and Medicine, Japan (Approval Number NCGM-G-003172). During the survey, the research assistants explained the study objectives and procedures and obtained written informed consent from all participants.

## 3. Results

Tables 1 and 2 show the basic characteristics of the study participants (*n* = 1102), according to alcohol consumption and cigarette smoking categories. The proportion of nonalcohol consumers was 27.6%, daily alcohol consumers < 1 drink per day was 32.2%, 1–1.9 drinks per day was 16.6%, and ≥ 2 standard drinks per day was 23.6%. The proportion of never smokers was 17.6%, 29.8% for former smokers, and 27.9% and 24.7% for current smokers consuming ≤ 10 or ≥ 11 cigarettes per day, respectively. Alcohol consumers were more likely to be former or current smokers, eat ≥ 1 serving per day of fruits and vegetables, practice ≥ 600 METs physical exercise, and have a BMI ≥ 23 kg/m^2^. Current cigarette smokers were more likely to be aged ≥ 45 years, have primary school or lower educational qualifications, and have a BMI < 23 kg/m^2^ than never smokers.

Table 3 and Table S1 show the association between alcohol consumption, cigarette smoking categories, and HOMA-beta and HOMA-IR. Alcohol consumption categories were negatively and significantly associated with HOMA-beta in a dose–response relationship in Model 3 (i.e., the main model): no drink (GM 71.5; 95% CI 65.3, 77.7), < 1 standard drink per day (GM 60.3; 95% CI 55.5, 65.1), 1–1.9 standard drink (GM 57.4; 95% CI 51.3, 63.6), and ≥ 2 standard drinks (GM 52.5; 95% CI 47.7, 57.3). This association remained consistent in Model 4. Cigarette smoking was not associated with HOMA-beta in Models 3 and 4. None of the alcohol consumer categories were associated with HOMA-IR when BMI was not adjusted in Model 3. However, in Model 4, adjusted for BMI, the highest alcohol consumer group (i.e., ≥ 2 standard drinks per day) showed a significantly lower HOMA-IR (GM 1.28; 95% CI 1.18, 1.38) than the no alcohol consumer group (GM 1.43; 95% CI 1.32, 1.54). In the smoking categories, current smokers < 10 cigarettes per day (GM 1.18; 95% CI 1.08, 1.27) or ≥ 11 cigarettes per day (GM 1.23; 95% CI 1.12, 1.34) showed significantly lower HOMA-IR than never smokers (GM 1.51; 95% CI 1.35, 1.67) in Model 3. However, these associations were attenuated and marginally significant in Model 4; current smokers < 10 cigarettes per day (GM 1.26; 95% CI 1.17, 1.35 [*p* = 0.09]) or ≥ 11 cigarettes per day (GM 1.25; 95% CI 1.15, 1.34 [*p* = 0.07]) showed marginally lower HOMA-IR than never smokers (GM 1.39, 95% CI 1.26, 1.52).

After confirming the presence of a suggestive level of effect modification by BMI (i.e., alcohol consumption [*p* for interaction = 0.12] and cigarette smoking [*p* for interaction = 0.67] for HOMA-beta and alcohol consumption [*p* for interaction = 0.14] and cigarette smoking [*p* for interaction = 0.02] for HOMA-IR), we conducted a series of stratified analyses using a BMI of 23 kg/m^2^ as cutoff value (Table 4 and Table S2). Alcohol consumption was strongly associated with HOMA-beta in the BMI < 23 group (*n* = 615) compared with the BMI ≥ 23 group (*n* = 487). Specifically, all alcohol consumer groups were negatively associated with HOMA-beta in the BMI < 23 group in Model 3: < 1 standard drink (GM 45.9; 95% CI 41.2, 50.7), 1–1.9 standard drink (GM 44.7; 95% CI 38.4, 51.0), and ≥ 2 standard drinks (GM 39.5; 95% CI 34.6, 44.3) compared with nonconsumers (GM 61.9; 95% CI 55.4, 68.5). In the BMI ≥ 23 group, only the group consuming ≥ 2.0 standard drinks per day (GM 72.2; 95% CI 64.9, 79.5) was associated with lower HOMA-beta compared with nonconsumers (GM 85.3, 95% CI 76.2, 94.4) in Model 3. However, smoking category was not associated with HOMA-beta in either BMI group.

For HOMA-IR, alcohol consumption was negatively associated with HOMA-IR in the BMI < 23 group in Model 3: < 1 standard drink per day (GM 0.95; 95% CI 0.86, 1.04) and ≥ 2 standard drinks per day (GM 0.90; 95% CI 0.80, 1.01) compared to nonconsumers (GM 1.09; 95% CI 0.99, 1.20), whereas alcohol consumption was not associated with HOMA-IR in the BMI ≥ 23 group. Smoking was not associated with HOMA-IR in either BMI group in Model 3.

For the associations with fasting glucose (Table S3), 1–1.9 standard drink (GM 99.0; 95% CI 96.3, 101.7) and ≥ 2 standard drinks (GM 100.3; 95% CI 97.9, 102.7) were significantly associated with higher fasting glucose compared to nonconsumers (GM 95.6; 95% CI 93.4, 97.8) in Model 3. The associations remained statistically significant in Model 4: 1–1.9 standard drinks (GM 99.1; 95% CI 96.5, 101.8) and ≥ 2 standard drinks (GM 100.1; 95% CI 97.7, 102.5) compared to nonconsumers (GM 95.7; 95% CI 93.5, 97.9). In the smoking categories, current smokers ≤ 10 cigarettes per day (GM 96.1; 95% CI 93.9, 98.3) or ≥ 11 cigarettes per day (GM 95.9; 95% CI 93.6, 98.2) showed significantly lower fasting glucose levels compared with never smokers (GM 99.3; 95% CI 96.6, 102.0) in Model 3. However, the associations were attenuated and became nonsignificant for current smokers ≤ 10 cigarettes per day (GM 96.6; 95% CI 94.4, 98.8) and marginally significant for current smokers ≥ 11 cigarettes per day (GM 96.0; 95% CI 93.7, 98.3), compared with never smokers (GM 98.6; 95% CI 96.0, 101.3) in Model 4.

## 4. Discussion

In this cross-sectional study of 1102 middle-aged men without a history of DM, we found that alcohol consumption was associated with reduced insulin secretion in both unadjusted and BMI-adjusted models. Cigarette smoking was associated with lower insulin resistance when BMI was unadjusted. However, this association became marginally significant after adjusting for BMI, potentially due to the mediating effect of BMI. In the stratified analyses, the associations of alcohol consumption with both insulin secretion and resistance were more pronounced in individuals with BMI < 23 kg/m^2^ than in those with BMI ≥ 23 kg/m^2^. Cigarette smoking was not associated with insulin secretion or resistance in the analysis stratified by BMI.

The inverse dose–response association between alcohol consumption and insulin secretion observed in this study corresponds to the results of the previous studies conducted in China, Korea, and Japan [8–12]. Notably, low insulin secretion capacity is an inherent characteristic of glucose metabolism in Asian populations [32] and may be more pronounced in individuals with a low BMI [12]. Our findings suggested that lower insulin capacity may be exacerbated by increased alcohol consumption. The negative effect of alcohol consumption on insulin secretion in a low BMI population should be given more attention in DM prevention efforts in Asian countries.

The lower insulin resistance observed in the highest alcohol consumption group after adjusting for BMI aligned with the results of the previous studies which showed that low-to-moderate alcohol consumption was associated with decreased insulin resistance [13–15]. In our study, the highest alcohol consumption group was defined as those consuming ≥ 2 standard drinks (about ≥ 20 g of ethanol) per day which was close to the peak of risk reduction (10–14 g) and lower than the threshold of risk increasing (63 g) identified in a previous meta-analysis study [4]. This may explain why lower insulin resistance was observed in alcohol consumers than in nonconsumers. Previous research has suggested that an increase in adiponectin concentration and anti-inflammatory substances induced by alcohol consumption improves insulin resistance [40–42]. Furthermore, in the stratified analysis, a significant inverse association between alcohol consumption and HOMA-IR was observed in participants with BMI < 23 kg/m^2^ but not in those with BMI ≥ 23 kg/m^2^. Although previous studies reported no apparent effect modification [9, 12, 13, 31], our findings suggest a potential effect modification by BMI on the association between alcohol consumption and insulin resistance.

Contrary to our hypothesis, which was based on experimental studies suggesting that toxic chemicals contained in cigarettes (e.g., nicotine and cadmium) impair pancreatic beta cells and suppress insulin secretion [43, 44], we found no association between smoking and insulin secretion. Previous epidemiological studies conducted in Italy (*n* = 2412 men aged 35–65 years) [21] and Japan (*n* = 411 men aged 40–78 years) [22] also reported no association. Although the reason for the inconsistency between our hypothesis and the findings from our study and two previous studies cannot be explained, it is possible that some of the other factors (e.g., genetic factors for nicotine metabolism [45] and secondhand smoke [22]) could impact the observed results.

Cigarette smoking was significantly associated with lower insulin resistance in Model 3, whereas the association became marginally significant after adjusting for BMI in Model 4. This change between the two models suggests that BMI acts as a mediator, as smokers tend to have lower BMI [26–28], which serves as a protective factor against insulin resistance. Four previous studies reported an association between cigarette smoking and lower insulin resistance. Two of these studies showed a significant association without adjustment for BMI but did not report results with adjustment for BMI [16, 21]. The other two studies showed a nonsignificant association before adjusting for BMI; one of these reported a marginally significant association after adjustment for BMI [17], while the other continued to show a nonsignificant association [25]. Due to a lack of consistent evidence from our study and these previous studies, we cannot fully explain the mechanism by which cigarette smoking is associated with lower insulin resistance, mediated by lower BMI. However, smoking is an established cause of DM [46], and our results do not imply that smoking is beneficial for improving insulin resistance.

This study had several limitations. First, our study sample did not fully represent middle-aged men in Vietnam because we did not employ a probability sampling procedure when recruiting study participants. Therefore, the findings of the present study should be interpreted with caution. Second, the prevalence of alcohol consumption and smoking may have been underestimated because self-reported data were used. However, the prevalence of cigarette smoking and alcohol consumption in this study was similar to that reported in a nationally representative survey using self-reported data [47]. Third, HOMA-beta and HOMA-IR are not direct measures of insulin secretion and resistance; however, previous studies have shown that HOMA estimation correlates with estimation using validated methods [48, 49]. Fourth, the cross-sectional nature of this study limits the ability to infer causality regarding the association of alcohol consumption and cigarette smoking with insulin secretion and resistance. Future studies should conduct longitudinal analyses to elucidate the long-term effects of alcohol consumption and smoking on insulin secretion and resistance.

## 5. Conclusions

In rural Vietnam, in middle-aged men without known DM, alcohol consumption was associated with low insulin secretion, which association remained consistent after adjusting for BMI. Cigarette smoking was associated with low insulin resistance in the unadjusted model, although this association became marginally significant after adjusting for BMI. These findings corresponded with higher fasting glucose levels observed in alcohol consumers and lower fasting glucose levels observed in smokers. Longitudinal studies are required to explore the effects of alcohol consumption and cigarette smoking on insulin secretion and resistance.

## Figures and Tables

**Table 1:** Basic characteristics of study participants according to alcohol consumption (*n* = 1102).

	**Non consumers**		**< 1 standard drink per day**		**1–1.9 standard drinks per day**		**≥ 2 standard drinks per day**	
	**n**	**(%)**	**n**	**(%)**	**n**	**(%)**	**n**	**(%)**
Overall	304	(27.6)	355	(32.2)	183	(16.6)	260	(23.6)
Age								
40–44	33	(10.9)	71	(20.0)	37	(20.2)	62	(23.8)
45–49	64	(21.1)	96	(27.0)	55	(30.1)	75	(28.8)
50–54	101	(33.2)	104	(29.3)	46	(25.1)	64	(24.6)
55–60	106	(34.9)	84	(23.7)	45	(24.6)	59	(22.7)
Education								
Primary school or lower	112	(36.8)	96	(27.0)	45	(24.6)	98	(37.7)
Secondary	123	(40.5)	139	(39.2)	62	(33.9)	94	(36.2)
Senior high or higher	69	(22.7)	120	(33.8)	76	(41.5)	68	(26.2)
Marital status								
Married	286	(94.1)	346	(97.5)	176	(96.2)	248	(95.4)
Not married	18	(5.9)	9	(2.5)	7	(3.8)	12	(4.6)
Occupation								
Government	17	(5.6)	48	(13.5)	31	(16.9)	31	(11.9)
Nongovernment	62	(20.4)	103	(29.0)	40	(21.9)	69	(26.5)
Self-employed	35	(11.5)	25	(7.0)	21	(11.5)	31	(11.9)
Farmer/fisherman	143	(47.0)	136	(38.3)	71	(38.8)	102	(39.2)
Unemployed/housework	23	(7.6)	27	(7.6)	13	(7.1)	13	(5.0)
Other	24	(7.9)	16	(4.5)	7	(3.8)	14	(5.4)
Household income by tertile								
Low	110	(36.2)	104	(29.3)	46	(25.1)	74	(28.5)
Middle	108	(35.5)	129	(36.3)	70	(38.3)	104	(40.0)
High	86	(28.3)	122	(34.4)	67	(36.6)	82	(31.5)
Parental history of diabetes								
Yes	32	(10.5)	48	(13.5)	15	(8.2)	39	(15.0)
No/unknown	272	(89.5)	307	(86.5)	168	(91.8)	221	(85.0)
Cigarette smoking (per day)								
Never	69	(22.7)	62	(17.5)	30	(16.4)	33	(12.7)
Former	75	(24.7)	124	(34.9)	55	(30.1)	74	(28.5)
Current ≤ 10 cigarettes	87	(28.6)	106	(29.9)	53	(29.0)	62	(23.8)
Current ≥ 11 cigarettes	73	(24.0)	63	(17.7)	45	(24.6)	91	(35.0)
Fruit and vegetable consumption (serving per day)								
< 1	69	(22.7)	41	(11.5)	29	(15.8)	38	(14.6)
1–1.9	123	(40.5)	124	(34.9)	60	(32.8)	92	(35.4)
2–2.9	62	(20.4)	92	(25.9)	48	(26.2)	62	(23.8)
≥ 3	50	(16.4)	98	(27.6)	46	(25.1)	68	(26.2)
Physical exercise								
< 600 METs	32	(10.5)	17	(4.8)	10	(5.5)	19	(7.3)
≥ 600 METs	272	(89.5)	338	(95.2)	173	(94.5)	241	(92.7)
BMI								
< 18.5	27	(8.9)	26	(7.3)	13	(7.1)	12	(4.6)
18.5–22.9	158	(52.0)	164	(46.2)	91	(49.7)	124	(47.7)
23.0–24.9	54	(17.8)	86	(24.2)	36	(19.7)	55	(21.2)
25.0–29.9	62	(20.4)	77	(21.7)	41	(22.4)	66	(25.4)
≥ 30.0	3	(1.0)	2	(0.6)	2	(1.1)	3	(1.2)

Abbreviations: BMI, body mass index; MET, metabolic equivalent of task.

**Table 2:** Basic characteristics of participants according to smoking status (*n* = 1102).

	**Never**		**Former**		**C** **u** **r** **r** **e** **n** **t** ≤ 10** cigarettes**		**C** **u** **r** **r** **e** **n** **t** ≥ 11** cigarettes**	
	**n**	**(%)**	**n**	**(%)**	**n**	**(%)**	**n**	**(%)**
Overall	194	(17.6)	328	(29.8)	308	(27.9)	272	(24.7)
Age								
40–44	58	(29.9)	42	(12.8)	50	(16.2)	53	(19.5)
45–49	55	(28.4)	81	(24.7)	82	(26.6)	72	(26.5)
50–54	45	(23.2)	104	(31.7)	92	(29.9)	74	(27.2)
55–60	36	(18.6)	101	(30.8)	84	(27.3)	73	(26.8)
Education								
Primary school or lower	50	(25.8)	83	(25.3)	110	(35.7)	108	(39.7)
Secondary	65	(33.5)	140	(42.7)	105	(34.1)	108	(39.7)
Senior high or higher	79	(40.7)	105	(32.0)	93	(30.2)	56	(20.6)
Marital status								
Married	185	(95.4)	317	(96.6)	293	(95.1)	261	(96.0)
Not married	9	(4.6)	11	(3.4)	15	(4.9)	11	(4.0)
Occupation								
Government	33	(17.0)	43	(13.1)	30	(9.7)	21	(7.7)
Nongovernment	43	(22.2)	78	(23.8)	87	(28.2)	66	(24.3)
Self-employed	26	(13.4)	40	(12.2)	26	(8.4)	20	(7.4)
Farmer/fisherman	67	(34.5)	127	(38.7)	123	(39.9)	135	(49.6)
Unemployed/housework	15	(7.7)	29	(8.8)	20	(6.5)	12	(4.4)
Other	10	(5.2)	11	(3.4)	22	(7.1)	18	(6.6)
Household income by tertile								
Low	53	(27.3)	96	(29.3)	95	(30.8)	90	(33.1)
Middle	72	(37.1)	115	(35.1)	123	(39.9)	101	(37.1)
High	69	(35.6)	117	(35.7)	90	(29.2)	81	(29.8)
Parental history of diabetes								
Yes	32	(16.5)	46	(14.0)	29	(9.4)	27	(9.9)
No/unknown	162	(83.5)	282	(86.0)	279	(90.6)	245	(90.1)
Alcohol drinking (standard drinking per day)								
No	69	(35.6)	75	(22.9)	87	(28.2)	73	(26.8)
< 1	62	(32.0)	124	(37.8)	106	(34.4)	63	(23.2)
1–1.9	30	(15.5)	55	(16.8)	53	(17.2)	45	(16.5)
≥ 2	33	(17.0)	74	(22.6)	62	(20.1)	91	(33.5)
Fruit and vegetable consumption (serving per day)								
< 1	33	(17.0)	33	(10.1)	52	(16.9)	59	(21.7)
1–1.9	66	(34.0)	101	(30.8)	127	(41.2)	105	(38.6)
2–2.9	49	(25.3)	95	(29.0)	61	(19.8)	59	(21.7)
≥ 3	46	(23.7)	99	(30.2)	68	(22.1)	49	(18.0)
Physical exercise								
< 600 METs	17	(8.8)	27	(8.2)	19	(6.2)	15	(5.5)
≥ 600 METs	177	(91.2)	301	(91.8)	289	(93.8)	257	(94.5)
BMI								
≤ 18.5	6	(3.1)	8	(2.4)	36	(11.7)	28	(10.3)
18.5–22.9	72	(37.1)	160	(48.8)	171	(55.5)	134	(49.3)
23.0–24.9	55	(28.4)	73	(22.3)	46	(14.9)	57	(21.0)
25.0–29.9	59	(30.4)	85	(25.9)	53	(17.2)	49	(18.0)
≥ 30.0	2	(1.0)	2	(0.6)	2	(0.6)	4	(1.5)

Abbreviations: BMI, body mass index; MET, metabolic equivalent of task.

**Table 3:** Associations between alcohol consumption, cigarette smoking, and HOMA-beta and HOMA-IR in 1102 men who participated in the Khanh Hoa Cardiovascular Study, Vietnam (2019–2020).

	**Model 1a**			**Model 1b**			**Model 2**			**Model 3**			**Model 4**		
	**Mean** ^c^	**(95% CI)**		**Mean** ^c^	**(95% CI)**		**Mean** ^c^	**(95% CI)**		**Mean** ^c^	**(95% CI)**		**Mean** ^c^	**(95% CI)**	
HOMA-beta															
Alcohol consumption^a^															
No	68.1	(62.6, 73.7)					69.5	(63.8, 75.2)		71.5	(65.3, 77.7)		72.5	(66.8, 78.2)	
< 1	61.3	(56.7, 65.9)	^†^				60.9	(56.3, 65.5)	⁣^∗^	60.3	(55.5, 65.1)	⁣^∗^	59.8	(55.5, 64.1)	⁣^∗^
1–1.9	59.1	(52.9, 65.3)	⁣^∗^				58.8	(52.7, 64.9)	⁣^∗^	57.4	(51.3, 63.6)	⁣^∗^	58.2	(52.4, 63.9)	⁣^∗^
≥ 2	53.0	(48.4, 57.7)	⁣^∗^				52.5	(47.9, 57.2)	⁣^∗^	52.5	(47.7, 57.3)	⁣^∗^	51.6	(47.3, 56.0)	⁣^∗^
Cigarette smoking^b^															
Never				66.9	(60.0, 73.7)		64.2	(57.6, 70.7)		61.9	(55.3, 68.4)		58.3	(52.6, 64.0)	
Former				64.1	(59.1, 69.2)		65.1	(60.0, 70.2)		63.9	(58.6, 69.3)		62.7	(58.0, 67.5)	
Current ≤ 10				55.1	(50.6, 59.6)	⁣^∗^	54.9	(50.5, 59.3)	⁣^∗^	55.7	(51.0, 60.4)		58.3	(53.8, 62.9)	
Current ≥ 11				58.9	(53.8, 63.9)	^†^	59.7	(54.6, 64.8)		62.0	(56.4, 67.6)		62.5	(57.4, 67.7)	
HOMA-IR															
Alcohol consumption															
No	1.37	(1.23, 1.51)					1.37	(1.24, 1.50)		1.41	(1.28, 1.53)		1.43	(1.32, 1.54)	
<1	1.33	(1.20, 1.46)					1.32	(1.20, 1.44)		1.31	(1.21, 1.41)		1.30	(1.21, 1.39)	^†^
1–1.9	1.36	(1.20, 1.53)					1.37	(1.21, 1.53)		1.35	(1.20, 1.49)		1.37	(1.25, 1.50)	
≥ 2	1.29	(1.15, 1.43)					1.31	(1.18, 1.44)		1.30	(1.18, 1.42)		1.28	(1.18, 1.38)	⁣^∗^
Cigarette smoking															
Never				1.57	(1.40, 1.75)		1.57	(1.39, 1.75)		1.51	(1.35, 1.67)		1.39	(1.26, 1.52)	
Former				1.54	(1.40, 1.69)		1.55	(1.40, 1.69)		1.51	(1.39, 1.64)		1.48	(1.38, 1.58)	
Current ≤ 10				1.16	(1.05, 1.27)	⁣^∗^	1.16	(1.05, 1.27)	⁣^∗^	1.18	(1.08, 1.27)	⁣^∗^	1.26	(1.17, 1.35)	^†^
Current ≥ 11				1.18	(1.07, 1.30)	⁣^∗^	1.19	(1.07, 1.30)	⁣^∗^	1.23	(1.12, 1.34)	⁣^∗^	1.25	(1.15, 1.34)	^†^

*Note:* Models 1a and 1b included alcohol consumption and cigarette smoking, respectively. Model 2 included the two exposure variables and age. Model 3 included education, occupation, household income, family history of diabetes, fruit and vegetable consumption, and physical activity levels in addition to the variables included in Model 2. Model 4 included the variables used in Model 3 and BMI.

^†^ *p* value < 0.10, ⁣^∗^*p* value < 0.05 (difference from the reference category).

^a^The amount of standard drinks consumed per day.

^b^The number of cigarettes smoked per day.

^c^Mean represents Geometric Mean.

**Table 4:** Associations between alcohol consumption, smoking status, and HOMA-beta and HOMA-IR in 1102 men who participated in the Khanh Hoa Cardiovascular Study, Vietnam (2019–2020), stratified by BMI at 23 kg/m^2^.

	**Model 1a**			**Model 1b**			**Model 2**			**Model 3**		
	**Geometric mean**	**(95% CI)**		**Geometric mean**	**(95% CI)**		**Geometric mean**	**(95% CI)**		**Geometric mean**	**(95% CI)**	
**BMI < 23** (***n*= 615**)													
HOMA-beta												
Alcohol consumption^a^												
No	59.5	(53.1, 65.8)					60.8	(54.2, 67.3)		61.9	(55.4, 68.5)	
< 1	46.6	(41.6, 51.5)	⁣^∗^				46.5	(41.6, 51.4)	⁣^∗^	45.9	(41.2, 50.7)	⁣^∗^
1–1.9	46.0	(39.4, 52.6)	⁣^∗^				45.4	(38.9, 51.8)	⁣^∗^	44.7	(38.4, 51.0)	⁣^∗^
≥ 2	40.0	(35.0, 45.0)	⁣^∗^				39.4	(34.4, 44.3)	⁣^∗^	39.5	(34.6, 44.3)	⁣^∗^
Cigarette smoking^b^												
Never				49.0	(40.7, 57.2)		46.8	(39.1, 54.6)		45.9	(38.4, 53.4)	
Former				51.0	(45.2, 56.9)		51.9	(46.1, 57.8)		49.1	(43.6, 54.7)	
Current ≤ 10				45.2	(40.5, 49.9)		45.1	(40.5, 49.6)		45.9	(41.3, 50.4)	
Current ≥ 11				49.6	(43.8, 55.4)		50.0	(44.2, 55.7)		52.3	(46.3, 58.2)	
HOMA-IR												
Alcohol consumption												
No	1.09	(0.97, 1.20)					1.09	(0.98, 1.20)		1.09	(0.99, 1.20)	
< 1	0.96	(0.86, 1.06)	^†^				0.95	(0.86, 1.05)	^†^	0.95	(0.86, 1.04)	⁣^∗^
1–1.9	0.97	(0.84, 1.10)					0.95	(0.83, 1.08)		0.95	(0.83, 1.07)	^†^
≥ 2	0.88	(0.78, 0.99)	⁣^∗^				0.90	(0.80, 1.00)	⁣^∗^	0.90	(0.80, 1.01)	⁣^∗^
Cigarette smoking												
Never				1.03	(0.88, 1.19)		1.02	(0.87, 1.18)		1.02	(0.87, 1.17)	
Former				1.23	(1.10, 1.36)	^†^	1.23	(1.10, 1.35)	⁣^∗^	1.19	(1.06, 1.31)	
Current ≤ 10				0.89	(0.80, 0.97)	^†^	0.89	(0.80, 0.97)		0.89	(0.81, 0.98)	
Current ≥ 11				0.86	(0.77, 0.95)	⁣^∗^	0.86	(0.77, 0.95)	^†^	0.89	(0.79, 0.98)	
**BMI ≥ 23** (***n*= 487**)													
HOMA-beta												
Alcohol consumption												
No	84.2	(75.5, 92.8)					84.2	(75.4, 93.1)		85.3	(76.2, 94.4)	
< 1	84.1	(76.8, 91.5)					83.7	(76.3, 91.1)		83.9	(76.6, 91.2)	
1–1.9	82.1	(71.8, 92.4)					82.3	(71.9, 92.7)		81.0	(70.8, 91.3)	
≥ 2	72.2	(65.0, 79.5)	⁣^∗^				72.6	(65.2, 80.0)	⁣^∗^	72.2	(64.9, 79.5)	⁣^∗^
Cigarette smoking												
Never				82.4	(73.8, 91.0)		81.5	(72.7, 90.2)		81.5	(72.8, 90.2)	
Former				81.5	(74.3, 88.8)		81.8	(74.5, 89.1)		81.6	(74.3, 88.9)	
Current ≤ 10				82.6	(73.4, 91.9)		82.0	(72.8, 91.2)		81.6	(72.5, 90.7)	
Current ≥ 11				75.7	(67.6, 83.8)		76.9	(68.6, 85.2)		77.4	(69.1, 85.8)	
HOMA-IR												
Alcohol consumption												
No	1.93	(1.72, 2.14)					1.89	(1.68, 2.10)		1.92	(1.71, 2.13)	
< 1	1.98	(1.79, 2.16)					1.97	(1.79, 2.16)		1.97	(1.79, 2.15)	
1–1.9	2.18	(1.89, 2.48)					2.20	(1.90, 2.50)	^†^	2.16	(1.87, 2.44)	
≥ 2	1.96	(1.75, 2.17)					2.00	(1.78, 2.21)		1.99	(1.78, 2.20)	
Cigarette smoking												
Never				2.10	(1.87, 2.33)		2.14	(1.90, 2.39)		2.11	(1.88, 2.35)	
Former				2.00	(1.81, 2.19)		1.98	(1.80, 2.17)		1.95	(1.77, 2.13)	
Current ≤ 10				1.99	(1.75, 2.22)		1.98	(1.74, 2.21)		2.00	(1.76, 2.23)	
Current ≥ 11				1.88	(1.67, 2.10)		1.87	(1.66, 2.08)		1.93	(1.72, 2.15)	

*Note:* Models 1a and 1b included alcohol consumption and cigarette smoking, respectively. Model 2 included the two exposure variables and age. Model 3 included education, occupation, household income, family history of diabetes, fruit and vegetable consumption, and physical activity levels in addition to the variables used in Model 2. When we examined the effect modification by BMI in relation to HOMA-beta, *p* for interaction was 0.12 for the interaction between BMI and alcohol consumption and 0.67 for that between BMI and cigarette smoking. The corresponding HOMA-IR was 0.14 for alcohol consumption and 0.02 for cigarette smoking.

^†^ *p* value < 0.10, ⁣^∗^*p* value < 0.05 (difference from the reference category).

^a^Standard drinks per day.

^b^The number of cigarettes smoked per day.

## Data Availability

The data are available upon reasonable request to the corresponding author.

## Supporting Information

Supporting Information Additional supporting information can be found online in the Supporting Information section. The supporting information for this article can be found online at the following: Table S1: Associations between alcohol consumption, cigarette smoking, and HOMA-beta and HOMA-IR in 1102 men who participated in the Khanh Hoa Cardiovascular Study, Vietnam (2019–2020). Table S2: Associations between alcohol consumption, smoking status, and HOMA-beta and HOMA-IR in 1102 men who participated in the Khanh Hoa Cardiovascular Study, Vietnam (2019–2020), stratified by BMI at 23 kg/m^2^. Table S3: Associations between alcohol consumption, cigarette smoking, and fasting glucose level in 1102 men who participated in the Khanh Hoa Cardiovascular Study, Vietnam (2019–2020).
