# Supplementary material for: Associations Between Alcohol Consumption and Cigarette Smoking and Insulin Secretion and Resistance in Vietnamese Men Without a History of Diabetes: A Cross-Sectional Study
Source: J Diabetes Res. 2025 Sep 24;2025:1068375. doi: 10.1155/jdr/1068375 (PMC12488309; doi:10.1155/jdr/1068375)
Supplement: Supporting Information — Additional supporting information can be found online in the Supporting Information section. The supporting information for this article can be found online at the following: Table S1: Associations between alcohol consumption, cigarette smoking, and HOMA-beta and HOMA-IR in 1102 men who participated in the Khanh Hoa Cardiovascular Study, Vietnam (2019–2020). Table S2: Associations between alcohol consumption, smoking status, and HOMA-beta and HOMA-IR in 1102 men who participated in the Khanh Hoa Cardiovascular Study, Vietnam (2019–2020), stratified by BMI at 23 kg/m2. Table S3: Associations between alcohol consumption, cigarette smoking, and fasting glucose level in 1102 men who participated in the Khanh Hoa Cardiovascular Study, Vietnam (2019–2020). [file 1068375.f1.docx]

# **Supplementary material**

# **Table S1.** Associations between alcohol consumption, cigarette smoking and HOMA-beta and HOMA-IR in 1,102 men who participated in the Khanh Hoa Cardiovascular Study, Vietnam (2019-2020)

|  | Model 1a | |  | Model 1b | |  | Model 2 | |  | Model 3 | |  | Model 4 | |
| --- | --- | --- | --- | --- | --- | --- | --- | --- | --- | --- | --- | --- | --- | --- |
|  | coeff | (95%CI) |  | coeff | (95%CI) |  | coeff | (95%CI) |  | coeff | (95%CI) |  | coeff | (95%CI) |
| **HOMA-beta** |  |  |  |  |  |  |  |  |  |  |  |  |  |  |
| **Alcohol consumption^a^** |  |  |  |  |  |  |  |  |  |  |  |  |  |  |
| No | ref |  |  |  |  |  | ref |  |  | ref |  |  | ref |  |
| <1 | -0.105 | (-0.216, 0.006) |  |  |  |  | -0.132 | (-0.244, -0.020) |  | -0.171 | (-0.282, -0.059) |  | -0.193 | (-0.298, -0.087) |
| 1-1.9 | -0.142 | (-0.275, -0.009) |  |  |  |  | -0.167 | (-0.300, -0.034) |  | -0.220 | (-0.352, -0.087) |  | -0.220 | (-0.345, -0.095) |
| ≥2 | -0.250 | (-0.370, -0.130) |  |  |  |  | -0.280 | (-0.402, -0.158) |  | -0.309 | (-0.429, -0.189) |  | -0.340 | (-0.453, -0.226) |
| **Cigarette smoking^b^** |  |  |  |  |  |  |  |  |  |  |  |  |  |  |
| Never |  |  |  | ref |  |  | ref |  |  | ref |  |  | ref |  |
| Former |  |  |  | -0.042 | (-0.171, 0.087) |  | 0.015 | (-0.115, 0.145) |  | 0.033 | (-0.095, 0.161) |  | 0.073 | (-0.049, 0.194) |
| Current ≤10 |  |  |  | -0.194 | (-0.324, -0.063) |  | -0.155 | (-0.286, -0.025) |  | -0.105 | (-0.234, 0.023) |  | 0.000 | (-0.123, 0.123) |
| Current ≥11 |  |  |  | -0.128 | (-0.262, 0.006) |  | -0.072 | (-0.206, 0.062) |  | 0.002 | (-0.131, 0.135) |  | 0.069 | (-0.057, 0.196) |
|  |  |  |  |  |  |  |  |  |  |  |  |  |  |  |
| **HOMA-IR** |  |  |  |  |  |  |  |  |  |  |  |  |  |  |
| **Alcohol consumption** |  |  |  |  |  |  |  |  |  |  |  |  |  |  |
| No | ref |  |  |  |  |  | ref |  |  | ref |  |  | ref |  |
| <1 | -0.028 | (-0.142, 0.086) |  |  |  |  | -0.037 | (-0.151, 0.076) |  | -0.070 | (-0.182, 0.042) |  | -0.095 | (-0.196, 0.005) |
| 1-1.9 | -0.006 | (-0.142, 0.131) |  |  |  |  | 0.003 | (-0.133, 0.138) |  | -0.042 | (-0.175, 0.091) |  | -0.041 | (-0.160, 0.079) |
| ≥2 | -0.061 | (-0.184, 0.062) |  |  |  |  | -0.043 | (-0.166, 0.081) |  | -0.075 | (-0.196, 0.045) |  | -0.114 | (-0.223, -0.005) |
| **Cigarette smoking** |  |  |  |  |  |  |  |  |  |  |  |  |  |  |
| Never |  |  |  | ref |  |  | ref |  |  | ref |  |  | ref |  |
| Former |  |  |  | -0.019 | (-0.149, 0.110) |  | -0.012 | (-0.145, 0.120) |  | 0.000 | (-0.128, 0.129) |  | 0.061 | (-0.055, 0.177) |
| Current ≤10 |  |  |  | -0.308 | (-0.439, -0.177) |  | -0.303 | (-0.435, -0.171) |  | -0.251 | (-0.380, -0.121) |  | -0.103 | (-0.221, 0.014) |
| Current ≥11 |  |  |  | -0.285 | (-0.418, -0.151) |  | -0.278 | (-0.414, -0.143) |  | -0.203 | (-0.337, -0.069) |  | -0.111 | (-0.232, 0.011) |

Note: Models 1a and 1b included alcohol consumption and cigarette smoking, respectively. Model 2 included the two exposure variables and age. Model 3 included education, occupation, household income, family history of diabetes, fruit and vegetable consumption, and physical activity levels in addition to the variables included in Model 2. Model 4 included the variables used in Model 3 and BMI. ^a^ the amount of standard drinks consumed per day; ^b^ the number of cigarettes smoked per day.

# **Table S2.** Associations between alcohol consumption, smoking status and HOMA- beta and HOMA-IR in 1,102 men who participated in the Khanh Hoa Cardiovascular Study, Vietnam (2019-2020), stratified by BMI at 23kg/m^2^

|  | **BMI<23 (n=615)** | | | | | | | | | | |
| --- | --- | --- | --- | --- | --- | --- | --- | --- | --- | --- | --- |
|  | Model 1a | |  | Model 1b | |  | Model 2 | |  | Model 3 | |
|  | coeff | (95%CI) |  | coeff | (95%CI) |  | coeff | (95%CI) |  | coeff | (95%CI) |
| **HOMA-beta** |  |  |  |  |  |  |  |  |  |  |  |
| **Alcohol consumption^a^** |  |  |  |  |  |  |  |  |  |  |  |
| No |  |  |  |  |  |  |  |  |  |  |  |
| <1 | -0.244 | (-0.395, -0.093) |  |  |  |  | -0.268 | (-0.419, -0.116) |  | -0.299 | (-0.449, -0.149) |
| 1-1.9 | -0.256 | (-0.435, -0.077) |  |  |  |  | -0.292 | (-0.472, -0.113) |  | -0.327 | (-0.504, -0.149) |
| ≥2 | -0.396 | (-0.561, -0.231) |  |  |  |  | -0.434 | (-0.600, -0.268) |  | -0.450 | (-0.615, -0.286) |
| **Cigarette smoking^b^** |  |  |  |  |  |  |  |  |  |  |  |
| Never |  |  |  |  |  |  |  |  |  |  |  |
| Former |  |  |  | 0.041 | (-0.163, 0.244) |  | 0.103 | (-0.098, 0.304) |  | 0.068 | (-0.131, 0.267) |
| Current ≤10 |  |  |  | -0.080 | (-0.278, 0.117) |  | -0.038 | (-0.232, 0.156) |  | -0.001 | (-0.192, 0.190) |
| Current ≥11 |  |  |  | 0.013 | (-0.192, 0.218) |  | 0.064 | (-0.138, 0.267) |  | 0.130 | (-0.071, 0.331) |
|  |  |  |  |  |  |  |  |  |  |  |  |
| **HOMA-IR** |  |  |  |  |  |  |  |  |  |  |  |
| **Alcohol consumption** |  |  |  |  |  |  |  |  |  |  |  |
| No |  |  |  |  |  |  |  |  |  |  |  |
| <1 | -0.126 | (-0.265, 0.012) |  |  |  |  | -0.133 | (-0.270, 0.004) |  | -0.143 | (-0.280, -0.005) |
| 1-1.9 | -0.117 | (-0.281, 0.048) |  |  |  |  | -0.132 | (-0.295, 0.030) |  | -0.142 | (-0.304, 0.020) |
| ≥2 | -0.210 | (-0.361, -0.058) |  |  |  |  | -0.190 | (-0.340, -0.039) |  | -0.192 | (-0.342, -0.042) |
| **Cigarette smoking** |  |  |  |  |  |  |  |  |  |  |  |
| Never |  |  |  |  |  |  |  |  |  |  |  |
| Former |  |  |  | 0.172 | (-0.009, 0.353) |  | 0.182 | (0.001, 0.364) |  | 0.153 | (-0.029, 0.335) |
| Current ≤10 |  |  |  | -0.154 | (-0.330, 0.021) |  | -0.144 | (-0.319, 0.031) |  | -0.130 | (-0.304, 0.044) |
| Current ≥11 |  |  |  | -0.184 | (-0.366, -0.003) |  | -0.171 | (-0.353, 0.012) |  | -0.140 | (-0.323, 0.044) |

**Table S2. (Continued)**

| **BMI**≥**23 (n=487)** | | | | | | | | | | | |
| --- | --- | --- | --- | --- | --- | --- | --- | --- | --- | --- | --- |
|  | Model 1a | |  | Model 1b | |  | Model 2 | |  | Model 3 | |
|  | coeff | (95%CI) |  | coeff | (95%CI) |  | coeff | (95%CI) |  | coeff | (95%CI) |
| **HOMA-beta** |  |  |  |  |  |  |  |  |  |  |  |
| **Alcohol consumption** |  |  |  |  |  |  |  |  |  |  |  |
| No |  |  |  |  |  |  |  |  |  |  |  |
| <1 | 0.000 | (-0.135, 0.134) |  |  |  |  | -0.006 | (-0.144, 0.132) |  | -0.016 | (-0.157, 0.124) |
| 1-1.9 | -0.025 | (-0.187, 0.138) |  |  |  |  | -0.023 | (-0.188, 0.142) |  | -0.051 | (-0.221, 0.118) |
| ≥2 | -0.153 | (-0.296, -0.009) |  |  |  |  | -0.149 | (-0.298, 0.000) |  | -0.167 | (-0.317, -0.017) |
| **Cigarette smoking** |  |  |  |  |  |  |  |  |  |  |  |
| Never |  |  |  |  |  |  |  |  |  |  |  |
| Former |  |  |  | -0.011 | (-0.148, 0.126) |  | 0.004 | (-0.138, 0.146) |  | 0.002 | (-0.140, 0.143) |
| Current ≤10 |  |  |  | 0.002 | (-0.151, 0.156) |  | 0.006 | (-0.151, 0.163) |  | 0.001 | (-0.155, 0.157) |
| Current ≥11 |  |  |  | -0.085 | (-0.235, 0.064) |  | -0.058 | (-0.211, 0.095) |  | -0.051 | (-0.204, 0.103) |
|  |  |  |  |  |  |  |  |  |  |  |  |
| **HOMA-IR** |  |  |  |  |  |  |  |  |  |  |  |
| **Alcohol consumption** |  |  |  |  |  |  |  |  |  |  |  |
| No |  |  |  |  |  |  |  |  |  |  |  |
| <1 | 0.022 | (-0.121, 0.166) |  |  |  |  | 0.044 | (-0.102, 0.191) |  | 0.027 | (-0.119, 0.174) |
| 1-1.9 | 0.122 | (-0.051, 0.295) |  |  |  |  | 0.153 | (-0.022, 0.329) |  | 0.117 | (-0.059, 0.293) |
| ≥2 | 0.014 | (-0.139, 0.167) |  |  |  |  | 0.056 | (-0.102, 0.214) |  | 0.034 | (-0.123, 0.191) |
| **Cigarette smoking** |  |  |  |  |  |  |  |  |  |  |  |
| Never |  |  |  |  |  |  |  |  |  |  |  |
| Former |  |  |  | -0.049 | (-0.194, 0.097) |  | -0.077 | (-0.227, 0.074) |  | -0.080 | (-0.228, 0.067) |
| Current ≤10 |  |  |  | -0.055 | (-0.217, 0.107) |  | -0.081 | (-0.248, 0.085) |  | -0.056 | (-0.219, 0.107) |
| Current ≥11 |  |  |  | -0.109 | (-0.268, 0.049) |  | -0.136 | (-0.299, 0.027) |  | -0.089 | (-0.249, 0.072) |

Note. Models 1a and 1b included alcohol consumption and cigarette smoking, respectively. Model 2 included the two exposure variables and age. Model 3 included education, occupation, household income, family history of diabetes, fruit and vegetable consumption, and physical activity levels in addition to the variables used in Model 2. ^a^ standard drinks per day; ^b^ the number of cigarettes smoked per day.

# **Table S3.** Associations between alcohol consumption, cigarette smoking and fasting glucose level in 1,102 men who participated in the Khanh Hoa Cardiovascular Study, Vietnam (2019-2020)

|  | Model 1a | | | Model 1b | | | Model 2 | | | Model 3 | | | Model 4 | | |
| --- | --- | --- | --- | --- | --- | --- | --- | --- | --- | --- | --- | --- | --- | --- | --- |
|  | geometric mean | (95%CI) |  | geometric mean | (95%CI) |  | geometric mean | (95%CI) |  | geometric mean | (95%CI) |  | geometric mean | (95%CI) |  |
| **Fasting glucose** |  |  |  |  |  |  |  |  |  |  |  |  |  |  |  |
| **Alcohol consumption** |  |  |  |  |  |  |  |  |  |  |  |  |  |  |  |
| No | 96.0 | (93.6, 98.5) |  |  |  |  | 95.6 | (93.4, 97.9) |  | 95.6 | (93.4, 97.8) |  | 95.7 | (93.5, 97.9) |  |
| <1 | 96.6 | (94.2, 98.9) |  |  |  |  | 96.5 | (94.3, 98.7) |  | 96.6 | (94.5, 98.7) |  | 96.5 | (94.4, 98.5) |  |
| 1-1.9 | 98.6 | (95.8, 101.5) | † |  |  |  | 98.8 | (96.1, 101.6) | * | 99.0 | (96.3, 101.7) | * | 99.1 | (96.5, 101.8) | * |
| ≥2 | 99.9 | (97.3, 102.5) | * |  |  |  | 100.4 | (97.9, 102.9) | * | 100.3 | (97.9, 102.7) | * | 100.1 | (97.7, 102.5) | * |
| **Cigarette smoking** |  |  |  |  |  |  |  |  |  |  |  |  |  |  |  |
| Never |  |  |  | 98.7 | (96.0, 101.5) |  | 99.5 | (96.7, 102.2) |  | 99.3 | (96.6, 102.0) |  | 98.6 | (96.0, 101.3) |  |
| Former |  |  |  | 99.7 | (97.3, 102.1) |  | 99.5 | (97.2, 101.8) |  | 99.3 | (97.1, 101.5) |  | 99.1 | (96.9, 101.3) |  |
| Current ≤10 |  |  |  | 95.8 | (93.5, 98.1) | * | 95.9 | (93.6, 98.1) | * | 96.1 | (93.9, 98.3) | * | 96.6 | (94.4, 98.8) |  |
| Current ≥11 |  |  |  | 96.2 | (93.7, 98.6) | † | 95.8 | (93.4, 98.1) | * | 95.9 | (93.6, 98.2) | * | 96.0 | (93.7, 98.3) | † |

Note: Models 1a and 1b included alcohol consumption and cigarette smoking, respectively. Model 2 included the two exposure variables and age. Model 3 included education, occupation, household income, family history of diabetes, fruit and vegetable consumption, and physical activity levels in addition to the variables included in Model 2. Model 4 included the variables used in Model 3 and BMI.

Difference from the reference category: †p-value <0.10, * p-value <0.05.

^a^ the amount of standard drinks consumed per day; ^b^ the number of cigarettes smoked per day.
